# Mononuclear cell therapy attenuates atherosclerosis in apoE KO mice

**DOI:** 10.1186/1476-511X-10-155

**Published:** 2011-09-06

**Authors:** Marcella L Porto, Leandro CF Lima, Thiago MC Pereira, Breno V Nogueira, Clarissa L Tonini, Bianca P Campagnaro, Silvana S Meyrelles, Elisardo C Vasquez

**Affiliations:** 1 Laboratory of Transgenes and Cardiovascular Control, Dept. Physiological Sciences, Health Sciences Center, Federal University of Espirito Santo, Vitoria, ES, Brazil; 2 Emescam College of Health Sciences, Vitoria, ES, Brazil

**Keywords:** cell therapy, atherosclerosis, apoE KO mice, EPC

## Abstract

**Background:**

Recent studies have highlighted the potential of cell therapy for atherosclerosis. The aim of this study was to evaluate the effects of mononuclear cell (MNC) therapy on the development of atherosclerotic lesions in the apolipoprotein E knockout (apoE KO) mouse.

**Methods:**

We investigated vascular lipid deposition, vascular remodeling, oxidative stress, and endothelial nitric oxide synthase (eNOS) expression in apoE KO mice treated with spleen MNCs isolated from *lacZ *transgenic mice (apoE KO-MNC) for 8 weeks compared to untreated control mice (apoE KO).

**Results:**

Histological analysis of aortas showed a significant reduction in the lipid deposition area in apoE KO-MNC mice compared to apoE KO mice (0.051 ± 0.004 *vs *0.117 ± 0.016 mm^2^, respectively, p < 0.01). In addition, vessel morphometry revealed that MNC therapy prevented the outward (positive) remodeling in apoE KO mice that is normally observed (apoE KO-MNC: 0.98 ± 0.07 *vs *apoE KO: 1.37 ± 0.09), using wild-type mice (C57BL/6J) as a reference. ApoE KO-MNC mice also have reduced production of superoxide anions and increased eNOS expression compared to apoE KO mice. Finally, immunohistochemistry analysis revealed a homing of endothelial progenitor cells (EPCs) in the aortas of apoE KO-MNC mice.

**Conclusion:**

MNC therapy attenuates the progression of atherosclerosis in the aortas of apoE KO mice. Our data provide evidence that the mechanism by which this attenuation occurs includes the homing of EPCs, a decrease in oxidative stress and an upregulation of eNOS expression.

## Background

Atherosclerosis is a chronic and degenerative disease of the wall of the large arteries and is a leading cause of mortality and morbidity [1,2]. Despite progress in the treatment and repair of this disease, researchers are continually challenged to develop new successful approaches. Therapy using stem/progenitor cells has emerged as an alternative to conventional treatments for atherosclerosis, as demonstrated by some positive results in experimental and clinical studies [3,4].

The apolipoprotein E knockout (apoE KO) mouse, developed two decades ago [5,6], has been used as an experimental model of atherosclerosis because it spontaneously develops hypercholesterolemia and atherosclerosis in a reproducible manner, similar to what is observed in humans. The apoE KO mouse is a particularly useful model as it offers a unique opportunity to evaluate the mechanisms involved in the development of atherosclerosis and new therapies for treatment of this disease.

One potential therapy for atherosclerosis uses mononuclear cells (MNCs), which contain a subpopulation of endothelial progenitor cells (EPCs) [7]. However, the beneficial effects of MNC therapy on atherosclerosis are still a subject of controversy in both humans [8,9] and experimental models of atherosclerosis [10,11]. In this regard, the main objective of the current study was to evaluate the effects of MNC therapy on vascular atherosclerotic lesions in apoE KO mice and to elucidate the mechanisms by which MNC therapy attenuates the progression of these lesions. We hypothesized that MNC therapy attenuates the progression of vascular atherosclerosis through the homing of EPCs, reducing reactive oxygen species (ROS), and increasing the expression of endothelial nitric oxide synthase (eNOS).

## Materials and methods

### Animals

ApoE KO female mice (24-week-old) were randomly divided into two groups: 1) an apoE KO control group (n = 8) and 2) an apoE KO group that received MNC therapy (apoE KO-MNC, n = 8). β-galactosidase (β-gal) (encoded by the *lacZ *gene) transgenic mice (12-week-old) were used as MNC donors. Animals were obtained from animal facilities at the Federal University of Espirito Santo. Six-month-old apoE KO mice were fed a cholesterol-rich diet (1.25% cholesterol) for 4 months and were housed separately in temperature-controlled rooms (22°C) under a 12 h light/dark cycle. All procedures were conducted in accordance with the institutional guidelines for animal research and the protocols were previously approved by the Institutional Ethics *Committee for Use of Animals (CEUA 003/2008)*.

### Isolation of mononuclear cells from spleen

*LacZ *transgenic mice were euthanized with sodium thiopental overdose (100 mg/kg, intraperitoneal injection). The spleens were removed, homogenized, and mixed with Dulbecco's Modified Eagle Medium (DMEM) to nourish the cells. The homogenate was then loaded onto a histopaque gradient. The layer containing MNCs was removed and resuspended in DMEM for future intravenous injections.

### Transfer of spleen mononuclear cells

For MNC transfusions, 1 × 10^6 ^MNCs were resuspended in 100 μL DMEM and administered by intravenous injections into the tail vein of apoE KO mice over a period of 2 months (1 injection per week for a total of 8 injections). Uninjected apoE KO mice served as a control group. We included a control group of a age matched apoE KO mice that received vehicle-only (DMEM) injections. To test the preventive effects of MNC therapy, we also administered MNC transfusions to younger (16-week-old) apoE KO mice that were fed a high-cholesterol diet for 2 months prior to the start of injections.

### Measurement of plasma cholesterol levels

A blood sample (200 μL) was collected by intracardiac puncture of each animal and the plasma total cholesterol was measured using a commercial colorimetric kit (Bioclin, Belo Horizonte, Brazil).

### Histological analysis of aortic root plaque

At the end of the experiments, each mouse was euthanized with sodium thiopental overdose (100 mg/kg, intraperitoneal injection) and the left ventricle was perfused with 0.1 M phosphate-buffered saline (PBS, pH 7.4) followed by a 4% formaldehyde solution at a pressure of 100 mmHg. The aortic root and a portion of the ascendant aorta were embedded in OCT compound and cross-sectioned on a cryostat (Jung CM1800; Leica, Wetzlar, Germany) at a thickness of 10 μm. For each animal, aorta cross-sections were mounted on gelatin-coated slides and stained with Oil-Red-O (Sigma-Aldrich, St. Louis, MO, USA) to detect neutral lipids.

### Morphometry

Images of the aorta were captured with a color video camera (VKC150, Hitachi, Tokyo, Japan) connected to a microscope (Olympus AX70, Olympus, Center Valley, PA, USA) and analyzed using a National Institute of Health (NIH) Image program. An examiner blinded to the experimental groups performed the image analysis to prevent any bias is the interpretation of the results. Using a 4 × objective, the vessel cross-sectional area (V_CSA_) and the lumen cross-sectional area were calculated. The vascular remodeling ratio was obtained by dividing each animal's V_CSA _by the average V_CSA _of wild-type C57BL/6J (C57) mice and each sample was scored for absence of remodeling (0.95-1.05), inward remodeling (< 0.95), or outward remodeling (> 1.05).

### Localization of donor MNCs

The remaining whole aorta was opened lengthwise and stained *en face *for donor MNC localization. Aortic samples were incubated for 12 h at 37°C in freshly prepared β-gal staining solution (pH 4.0) containing 2.4 mM 5-bromo-4-chloro-3-indolyl-D-galactopyranoside (X-gal, Sigma Aldrich), 4.7 mmol/L potassium ferrocyanide, 4.9 mmol/L potassium ferricyanide, 150 mmol/L NaCl, 1 mmol/L MgCl_2 _and 40 mmol/L citric acid.

### Detection of superoxide production

Unfixed frozen sections of aorta were incubated in 2 μmol dihydroethidium (DHE) modified Kreb's solution containing 20 mmol of HEPES for 30 min in a light-protected chamber at 37°C.

### Immunohistochemistry

Cross-sections (10 μm) were cut on a cryostat and placed on gelatin-coated slides. Sections were air-dried and then slides were fixed for 20 minutes in acetone at -20°C. Slides were incubated with the following primary antibodies overnight: eNOS, 1:50 (BD Pharmingen, San Diego, CA, USA); vascular endothelial grown factor receptor (Flk-1), 1:50 (Abcam, Cambridge, MA, USA); hematopoietic stem cell antigen (CD133), 1:50 (Millipore, Billerica, MA, USA). A Vectastain ABC Elit kit was used for detection, with DAB or NovaRED staining (Vector Laboratories, Burlingame, CA, USA).

### Statistical Analysis

Data are presented as the mean ± SEM. Statistical analysis was performed with Student's *t*-test for independent samples. One-way analysis of variance (ANOVA), followed by the Tukey *post hoc *test for multiple comparisons, was used when appropriate. Statistical significance was set at p < 0.05.

## Results

Plasma total cholesterol levels of 24-week-old animals that were fed a high-cholesterol diet for 16 weeks was similar between apoE KO control mice and apoE KO mice that received MNC therapy for 8 weeks (1176 ± 213 *vs*. 1241 ± 167 mg/dL; p > 0.05).

**Figure 1:**
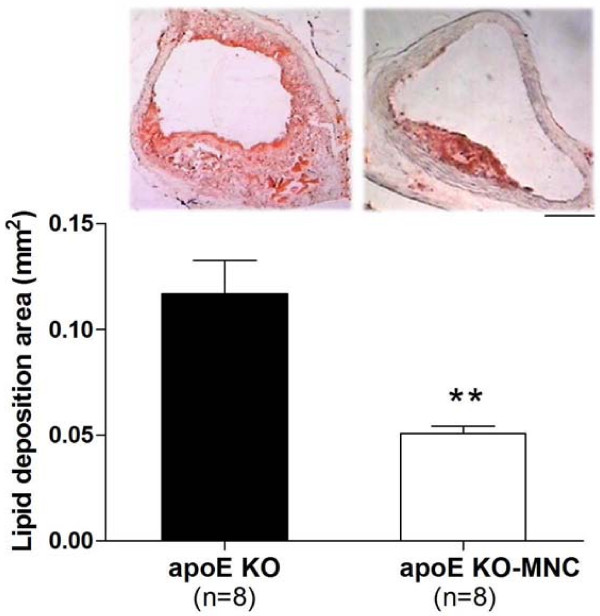
**Effect of MNC therapy on lipid deposition in aortas of apoE KO mice**. Top panel are typical photomicrographs of aorta root cross-sections comparing an apoE KO-MNC aorta to an apoE KO control aorta. (Oil-Red-O stain, original magnification × 4. Bar: 100 μm). Bar graph shows the average lipid deposition area comparing apoE KO-MNC to apoE KO group. Values are means ± SEM. **p < 0.01 compared to apoE KO (Student's *t *test).

Figure 1 shows the effect of MNC therapy on vessel lipid deposition. As shown by the histological analysis of a typical aortic root cross-section, apoE KO-MNC mice demonstrated marked reduction of the atherosclerotic lesion. A large lipid deposition area was observed in the aorta roots of 24-week-old apoE KO control and vehicle-only mice (0.117 ± 0.016 mm^2 ^and 0.109 ± 0.012 mm^2^, respectively). In contrast, the lipid deposition area was significantly reduced in approximately 45% (p < 0.01) of age-matched apoE KO-MNC mice (bar graph, Figure 1). This value of apoE KO-MNC was statistically similar to that observed in 16-week-old apoE KO mice (0.051 ± 0.004 vs. 0.049 ± 0.005 mm^2^, respectively), indicating that MNCs have atheroprotective properties.

**Figure 2:**
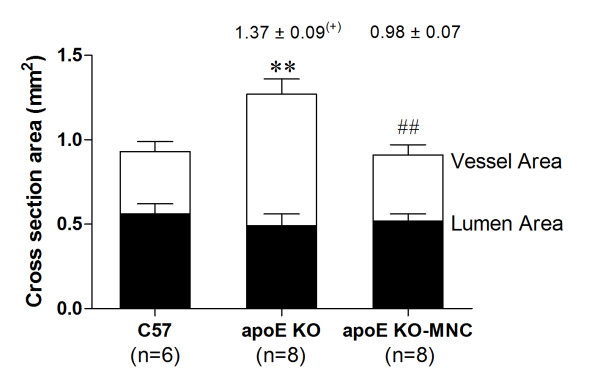
**Effect of MNC therapy on vessel and lumen cross-sectional areas**. Values are means ± SEM. **p < 0.01 compared to wild-type C57 vessel area; ^##^p < 0.01 compared to the apoE KO vessel area (one-way ANOVA). Numbers above the bars indicate the remodeling ratio using the C57 group as reference value and (+) indicates a positive (outward) remodeling.

Figure 2 summarizes data obtained from measurements of the aorta cross-sectional area and vascular remodeling in apoE KO and apoE KO-MNC mice, using wild-type C57 animals as reference values. The vessel lumen area was statistically similar in all groups analyzed. However, the vessel wall area was significantly increased in apoE KO mice (0.79 ± 0.05 mm^2^) compared to C57 mice (0.37 ± 0.05 mm^2^, p < 0.01). This increase was subsequently normalized by MNC therapy (apoE KO-MNC: 0.39 ± 0.04 mm^2^, p < 0.01). Consistent with this finding, the positive (> 1.05) vascular remodeling observed in apoE KO mice was prevented by MNC therapy (ratios of 1.37 ± 0.09 in apoE KO *vs*. 0.98 ± 0.07 in apoE KO-MNC).

**Figure 3:**
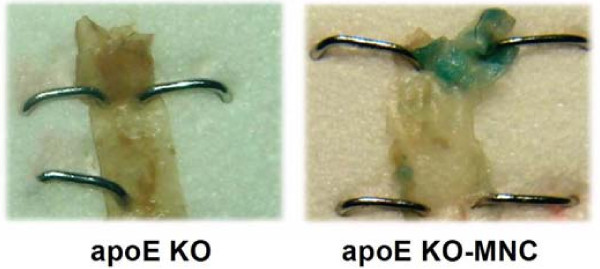
**Typical photograph of an aorta root stained *en face *showing β-gal-positive cells (blue) derived from *lacZ *mice in the apoE KO-MNC mouse, compared to the lack of staining in the apoE KO mouse**.

Figure 3 shows typical *en face *aortas from apoE KO and apoE KO-MNC mice that were stained with β-gal to identify donor-derived MNCs. All apoE KO animals that received MNC therapy from *lacZ *transgenic mice expressed β-gal staining in the aorta roots when compared with untreated and vehicle-only mice (data not shown), which lacked any vessel β-gal staining.

**Figure 4:**
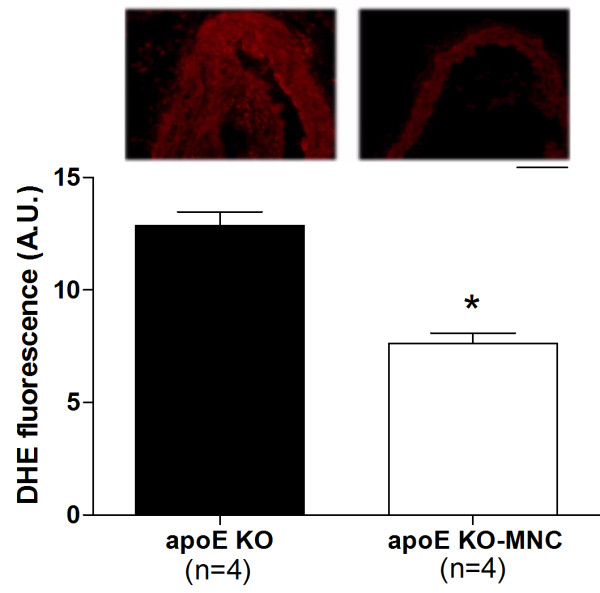
**Effect of MNC therapy on superoxide anion production in the aorta of apoE KO mice**. Top panel contains representative cross-sections stained with dihydroethidium (DHE) showing a bright ethidium fluorescence (red) in the apoE KO mouse compared to the apoE KO-MNC mouse (original magnification × 20. Bar: 50 μm). Bar graph shows average DHE fluorescence (AU: arbitrary units) comparing apoE KO to apoE KO-MNC mice. Values are means ± SEM. *p < 0.05 compared to apoE KO, Student's *t *test).

**Figure 5:**
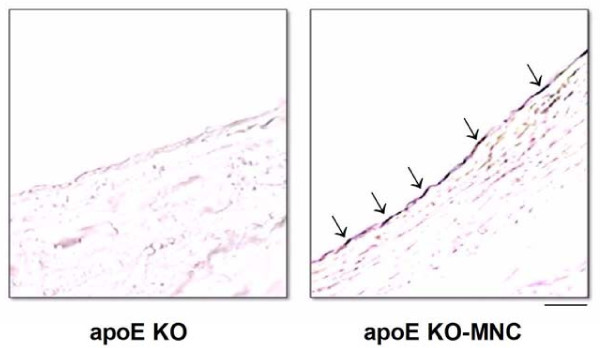
**Effect of MNC therapy on eNOS protein production in aortas of apoE KO mice**. Representative microphotographs reveal a positive immunoreaction (brown precipitates, indicated by arrow) for eNOS in the endothelium of the apoE KO-MNC mouse compared to the apoE KO mouse. (DAB stain, original magnification × 20. Bar: 50 μm).

**Figure 6:**
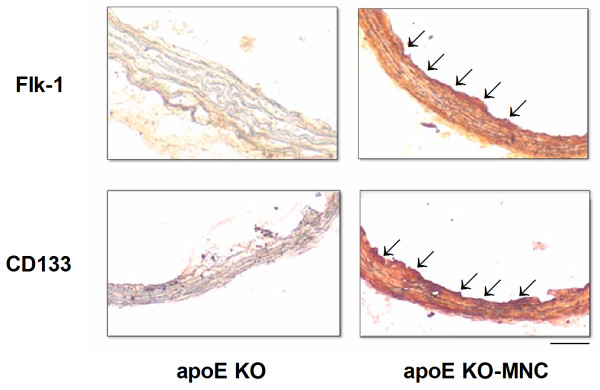
**Homing of endothelial progenitor cells after MNC therapy in apoE KO mice**. Photomicrographs are typical aorta cross-sections stained for the markers Flk-1 (vascular endothelial growth factor receptor) and CD133 (hematopoietic stem cell antigen) showing an intense immune reaction (red precipitate) in MNC-treated animals. (original magnification × 20. Bar: 50 μm).

To investigate the mechanisms by which MNC therapy reduced atherosclerosis, we evaluated superoxide (O_2_^·-^) production, eNOS production, and homing of endothelial progenitor cells. As illustrated in the microphotographs in Figure 4 (top panel), a dihydroethidium oxidative assay revealed intense ethidium fluorescence in the apoE KO mouse but not in the apoE KO-MNC mouse. On average, the aortas from apoE KO-MNC mice exhibited approximately 60% less ethidium fluorescence than untreated apoE KO mice (Figure 4, bottom panel). In addition, qualitative immunohistochemistry in aortas from apoE KO-MNC revealed increased expression of eNOS compared to untreated apoE KO mice (Figure 5). Homing of endothelial progenitor cells was confirmed by immunohistochemistry detection of Flk-1 and CD133, markers of endothelial progenitor cells, which showed a marked increase in Flk-1 and CD133 staining in the aorta endothelium of apoE KO-MNC mice compared to untreated apoE KO mice (illustrations in Figure 6).

## Discussion

In the present study we observed that 8 weeks of MNC therapy resulted in the attenuation of atherosclerotic lesions in aortas from apoE KO mice through the homing of EPCs, a reduction in the production of superoxide anions and an increase in eNOS expression.

Stem/progenitor cells, a subpopulation of MNCs, have unique characteristics that make them ideal for therapeutic purposes. They are undifferentiated, unspecialized, and can divide symmetrically and asymmetrically for long periods [12-14]. In the present study we observed that MNCs have atheroprotective properties in an apoE KO murine model of atherosclerosis, even under conditions of high total cholesterol in the plasma. The development of atherosclerosis was accelerated by a high-cholesterol diet; however, continuous treatment with MNCs derived from young *lacZ *transgenic mice caused a marked attenuation of the atherosclerotic lesion, without affecting the high total cholesterol levels in the plasma. This finding is in agreement with other studies in aortas [10,15] and carotid arteries [16] from murine models of atherosclerosis but conflicts with one study which found no significant effects from MNC therapy on atherosclerosis [17] and two studies which observed opposite results to ours [11,18]. This discrepancy could be due to the different methods used in these studies. For example, the study that found that there was no benefit from MNC therapy [17] treated the animals with fewer injections of MNCs (1 to 3 injections at similar concentrations) than did we and others [10,15] who observed a beneficial effect (6 to 8 injections). Other variable factors include the animal's age and gender.

The vascular remodeling of large arteries is considered an adaptive structural change that occurs in response to a variety of conditions, including atherosclerosis [19,20]. Our finding of a positive (outward) remodeling in aortas from apoE KO control mice corroborates this adaptive morphological change and is in agreement with previous studies [21-23]. A novel finding of this study revealed that animals that received MNC therapy (apoE KO-MNC mice) did not show vascular positive remodeling, most likely as a direct consequence of marked attenuation of the atherosclerotic process.

The X-gal *en face *analysis of aortas from apoE KO mice revealed the presence of donor MNCs (from a *lacZ *mouse) in atherosclerotic areas, indicating that the therapeutic effect of MNCs is at the level of the arterial wall, as has been described by others [10,24,25]. We then investigated the possible mechanisms by which MNCs could locally mediate the attenuation of atherosclerosis in apoE KO mice.

It is known that the number of circulating EPCs is inversely proportional to the risk of cardiovascular diseases [26], suggesting that increasing the number of circulating EPCs could be a powerful therapy in the treatment of atherosclerosis [10,27]. Based on what has been done in other studies [28], we used the spleen as a source of EPCs, which are contained within the mononuclear fraction. Our hypothesis that EPCs derived from the MNC fraction are involved in the anti-atherogenic response is based on our discovery of vascular homing of EPCs and the presence of markers Flk-1 and CD133 in aortas from apoE KO mice treated with MNCs [29,30]. As shown in Figure 6, aorta cross-sections from apoE KO-MNC mice showed intense expression of EPC markers. This indicates that in the presence of EPCs, the vessel wall is capable of accelerating the re-endothelialization and inhibition of neointimal formation. This mechanism of action by EPCs has also been demonstrated by others [31].

Clinical and experimental studies of atherosclerosis, including those using the apoE KO mouse model, support the concept that overabundance of ROS and/or a decline in antioxidant ability plays a causal role in atherosclerosis [32,33]. Moreover, the integrity of eNOS/NO production in the vasculature is critical for normal vascular function [34]. The effects of cell therapy on ROS production have not previously been studied in experimental atherosclerosis. To address this, in the present study we tested the hypothesis that the beneficial effects of MNC includes a reduction in oxidative stress. We found that MNC therapy in apoE KO mice resulted in a marked decrease in O_2_^·- ^production and a concomitant upregulation of eNOS in the aorta, supporting the idea that another important mechanism by which MNC therapy attenuates the progression of atherosclerosis is by relieving oxidative stress. Thus, we speculate that the increased number of circulating EPCs provided by MNC therapy upregulated the NO pathway. This finding is consistent with other studies that observed similar results in a model of experimental diabetes [35].

In conclusion, we have shown that MNC therapy attenuates atherosclerotic lesions in aortas from apoE KO mice. Our data provide evidence that the mechanisms by which MNC therapy is atheroprotective include homing of EPCs, reducing O_2_^·- ^production and upregulating eNOS expression. Although further studies are needed to reveal additional mechanisms underlying the atherosclerotic process in this murine model, the present data provide important evidence on the beneficial effect of cell therapy on atherosclerosis.

## Competing interests

The authors declare that they have no competing interests.

## Authors' contributions

MLP carried out experimental analysis and acquisition of data, analysis and interpretation of the data and drafted the manuscript. LCF carried out the experimental analysis. TMCP performed the statistics analysis and helped to draft the manuscript. BVN contributed to histology. CLT and BPC participated in the study's design and supervision and in the critical revision of the manuscript. SSM and ECV contributed to the conception, design and supervision of the study and interpretation of data. All authors read and approved the final version of the manuscript.

## References

[B1] Ross R Rous-Whipple Award Lecture: Atherosclerosis: a defense mechanism gone awry Am J Pathol 1993 143 4 987 1002 8214014 PMC1887075

[B2] Libby P Ridker PM Hansson GK Progress and challenges in translating the biology of atherosclerosis Nature 2011 473 7347 317 25 10.1038/nature10146 21593864

[B3] Chade AR Zhu X Lavi R Krier JD Pislaru S Simari RD Napoli C Lerman A Lerman LO Endothelial progenitor cells restore renal function in chronic experimental renovascular disease Circulation 2009 119 4 547 57 10.1161/CIRCULATIONAHA.108.788653 19153272 PMC2758066

[B4] Dotsenko O Stem/Progenitor cells, atherosclerosis and cardiovascular regeneration Open Cardiovasc Med J 2010 4 97 104 20386616 10.2174/1874192401004020097 PMC2852123

[B5] Plump AS Smith JD Hayek T Aalto-Setälä K Walsh A Verstuyft JG Rubin EM Breslow JL Severe hypercholesterolemia and atherosclerosis in apolipoprotein E-deficient mice created by homologous recombination in ES cells Cell 1992 71 2 343 53 10.1016/0092-8674(92)90362-G 1423598

[B6] Piedrahita JA Zhang SH Hagaman JR Oliver PM Maeda N Generation of mice carrying a mutant apolipoprotein E gene inactivated by gene targeting in embryonic stem cells Proc Natl Acad Sci USA 1992 89 10 4471 5 10.1073/pnas.89.10.4471 1584779 PMC49104

[B7] Zampetaki A Kirton JP Xu Q Vascular repair by endothelial progenitor cells Cardiovasc Res 2008 78 3 413 21 10.1093/cvr/cvn081 18349136

[B8] van Ramshorst J Bax JJ Beeres SL Dibbets-Schneider P Roes SD Stokkel MP de Roos A Fibbe WE Zwaginga JJ Boersma E Schalij MJ Atsma DE Intramyocardial bone marrow cell injection for chronic myocardial ischemia: a randomized controlled trial JAMA 2009 301 19 1997 2004 10.1001/jama.2009.685 19454638

[B9] Hopp E Lunde K Solheim S Aakhus S Arnesen H Forfang K Edvardsen T Smith HJ Regional myocardial function after intracoronary bone marrow cell injection in reperfused anterior wall infarction - a cardiovascular magnetic resonance tagging study J Cardiovasc Magn Reson 2011 13 22 10.1186/1532-429X-13-22 21414223 PMC3068099

[B10] Rauscher FM Goldschmidt-Clermont PJ Davis BH Wang T Gregg D Ramaswami P Pippen AM Annex BH Dong C Taylor DA Aging, progenitor cell exhaustion, and atherosclerosis Circulation 2003 108 4 457 63 10.1161/01.CIR.0000082924.75945.48 12860902

[B11] Silvestre JS Gojova A Brun V Potteaux S Esposito B Duriez M Clergue M Le Ricousse-Roussanne S Barateau V Merval R Groux H Tobelem G Levy B Tedgui A Mallat Z Transplantation of bone marrow-derived mononuclear cells in ischemic apolipoprotein E-knockout mice accelerates atherosclerosis without altering plaque composition Circulation 2003 108 23 2839 42 10.1161/01.CIR.0000106161.43954.DF 14656923

[B12] Yagita Y Kitagawa K Ohtsuki T Takasawa Ki Miyata T Okano H Hori M Matsumoto M Neurogenesis by progenitor cells in the ischemic adult rat hippocampus Stroke 2001 32 8 1890 6 10.1161/01.STR.32.8.1890 11486122

[B13] Yang Z Di Santo S Kalka C Current developments in the use of stem cell for therapeutic neovascularisation: is the future therapy "cell-free"? Swiss Med Wkly 2010 140 w13130 21170763 10.4414/smw.2010.13130

[B14] Przybycień K Kornacewicz Jach Z Machaliński B Stem cells in cardiological clinical trials Kardiol Pol 2011 69 6 601 9 Polish 21678304

[B15] Nelson WD Zenovich AG Ott HC Stolen C Caron GJ Panoskaltsis-Mortari A Barnes SA Xin X Taylor DA Sex-dependent attenuation of plaque growth after treatment with bone marrow mononuclear cells Circulation 2007 101 12 1319 27 10.1161/CIRCRESAHA.107.155564 17947799

[B16] Werner N Priller J Laufs U Endres M Böhm M Dirnagl U Nickenig G Bone marrow-derived progenitor cells modulate vascular reendothelialization and neointimal formation: effect of 3-hydroxy-3-methylglutaryl coenzyme a reductase inhibition Arterioscler Thromb Vasc Biol 2002 22 10 1567 72 10.1161/01.ATV.0000036417.43987.D8 12377731

[B17] Wassmann S Werner N Czech T Nickenig G Improvement of endothelial function by systemic transfusion of vascular progenitor cells Circulation 2006 99 8 e74 83 10.1161/01.RES.0000246095.90247.d4 16990568

[B18] George J Afek A Abashidze A Shmilovich H Deutsch V Kopolovich J Miller H Keren G Transfer of endothelial progenitor and bone marrow cells influences atherosclerotic plaque size and composition in apolipoprotein E knockout mice Arterioscler Thromb Vasc Biol 2005 25 12 2636 41 10.1161/01.ATV.0000188554.49745.9e 16195475

[B19] Glagov S Weisenberg E Zarins CK Stankunavicius R Kolettis GJ Compensatory enlargement of human atherosclerotic coronary arteries N Engl J Med 1987 316 22 1371 5 10.1056/NEJM198705283162204 3574413

[B20] Langille BL Arterial remodeling: relation to hemodynamics Can J Physiol Pharmacol 1996 74 7 834 41 10.1139/y96-082 8946070

[B21] Lutgens E de Muinck ED Heeneman S Daemen MJ Compensatory enlargement and stenosis develop in apoE(-/-) and apoE*3-Leiden transgenic mice Arterioscler Thromb Vasc Biol 2001 21 8 1359 65 10.1161/hq0801.093669 11498466

[B22] Nogueira BV Peotta VA Meyrelles SS Vasquez EC Evaluation of aortic remodeling in apolipoprotein E-deficient mice and renovascular hypertensive mice Arch Med Res 2007 38 8 816 21 10.1016/j.arcmed.2007.06.005 17923260

[B23] Pereira TM Nogueira BV Lima LC Porto ML Arruda JA Vasquez EC Meyrelles SS Cardiac and vascular changes in elderly atherosclerotic mice: the influence of gender Lipids Health Dis 2010 9 87 10.1186/1476-511X-9-87 20723257 PMC2936359

[B24] Hu Y Mayr M Metzler B Erdel M Davison F Xu Q Both donor and recipient origins of smooth muscle cells in vein graft atherosclerotic lesions Circulation 2002 91 7 e13 20 10.1161/01.RES.0000037090.34760.EE 12364395

[B25] Foteinos G Hu Y Xiao Q Metzler B Xu Q Rapid endothelial turnover in atherosclerosis-prone areas coincides with stem cell repair in apolipoprotein E-deficient mice Circulation 2008 117 14 1856 63 10.1161/CIRCULATIONAHA.107.746008 18378610

[B26] Schmidt-Lucke C Fichtlscherer S Aicher A Tschöpe C Schultheiss HP Zeiher AM Dimmeler S Quantification of circulating endothelial progenitor cells using the modified ISHAGE protocol PLoS One 2010 5 11 e13790 10.1371/journal.pone.0013790 21072182 PMC2972200

[B27] Strehlow K Werner N Berweiler J Link A Dirnagl U Priller J Laufs K Ghaeni L Milosevic M Böhm M Nickenig G Estrogen increases bone marrow-derived endothelial progenitor cell production and diminishes neointima formation Circulation 2003 107 24 3059 65 10.1161/01.CIR.0000077911.81151.30 12810616

[B28] Patschan D Krupincza K Patschan S Zhang Z Hamby C Goligorsky MS Dynamics of mobilization and homing of endothelial progenitor cells after acute renal ischemia: modulation by ischemic preconditioning Am J Physiol Renal Physiol 2006 291 1 F176 85 10.1152/ajprenal.00454.2005 16478972

[B29] Purpura KA George SH Dang SM Choi K Nagy A Zandstra PW Soluble Flt-1 regulates Flk-1 activation to control hematopoietic and endothelial development in an oxygen-responsive manner Stem Cells 2008 26 11 2832 42 10.1634/stemcells.2008-0237 18772315

[B30] Corbeil D Röper K Weigmann A Huttner WB AC133 hematopoietic stem cell antigen: human homologue of mouse kidney prominin or distinct member of a novel protein family? Blood 1998 91 7 2625 6 9516170

[B31] Umemura T Higashi Y Endothelial progenitor cells: therapeutic target for cardiovascular diseases J Pharmacol Sci 2008 108 1 1 6 10.1254/jphs.08R01CP 18776710

[B32] Kawashima S Malfunction of vascular control in lifestyle-related diseases: endothelial nitric oxide (NO) synthase/NO system in atherosclerosis J Pharmacol Sci 2004 96 4 411 9 10.1254/jphs.FMJ04006X6 15613778

[B33] Kunitomo M Oxidative stress and atherosclerosis Yakugaku Zasshi 2007 127 12 1997 2014 10.1248/yakushi.127.1997 18057788

[B34] Davignon J Ganz P Role of endothelial dysfunction in atherosclerosis Circulation 2004 109 23 Suppl 1 III27 32 15198963 10.1161/01.CIR.0000131515.03336.f8

[B35] Sambuceti G Morbelli S Vanella L Kusmic C Marini C Massollo M Augeri C Corselli M Ghersi C Chiavarina B Rodella LF L'Abbate A Drummond G Abraham NG Frassoni F Diabetes impairs the vascular recruitment of normal stem cells by oxidant damage, reversed by increases in pAMPK, heme oxygenase-1, and adiponectin Stem Cells 2009 27 2 399 407 10.1634/stemcells.2008-0800 19038792 PMC2729677

